# Genetic Alterations in Papillary Thyroid Carcinoma With Hashimoto^’^s Thyroiditis： *ANK3*, an Indolent Maintainer of Papillary Thyroid Carcinoma

**DOI:** 10.3389/fonc.2022.894786

**Published:** 2022-05-12

**Authors:** Chao Zeng, Jiali Long, Chunmiao Deng, Linying Xie, Hongmei Ma, Yimin Guo, Shuguang Liu, Min Deng

**Affiliations:** ^1^ Department of Pathology, The Eighth Affiliated Hospital, Sun Yat-sen University, Shenzhen, China; ^2^ Affiliated Cancer Hospital & Institute of Guangzhou Medical University, Guangzhou, China

**Keywords:** papillary thyroid carcinoma, SNV, ANK3, EMT, E-cadherin

## Abstract

Hashimoto’s thyroiditis (TH) is a risk factor for the occurrence of papillary thyroid carcinoma (PTC), which is considered to be the most common type of thyroid cancer. In recent years, the prevalence of PTC with TH has been increasing, but little is known about the genetic alteration in PTC with TH. This study analyzed the mutation spectrum and mutation signature of somatic single nucleotide variants (SNV) for 10 non-tumor and tumor pair tissues of PTC with TH using whole-exome sequencing. The ANK3 protein expression was evaluated by immunohistochemistry in PTC with TH and PTC samples. Moreover, the functional role of ANK3 in PTC cells was determined by CCK-8 proliferation assay, colony formation assays, cell cycle analysis, cell invasion and migration and *in vivo* study through overexpression assay. Our results showed three distinct mutational signatures and the C>T/G>A substitution was the most common type of SNV. Gene-set enrichment analysis showed that most of the significantly mutated genes were enriched in the regulation of actin cytoskeleton signaling. Moreover, *NCOR2, BPTF, ANK3*, and *PCSK5* were identified as the significantly mutated genes in PTC with TH, most of which have not been previously characterized. Unexpectedly, it was found that ANK3 was overexpressed in cytoplasm close to the membrane of PTC cells with TH and in almost all PTC cases, suggesting its role as a diagnostic marker of PTC. Ectopic expression of *ANK3* suppressed invasion and migration, increased apoptosis of B-CPAP and TPC-1 cells. Moreover, our findings revealed that enhanced *ANK3* expression inhibits growth of PTC cells both *in vitro* and *in vivo*. Ectopic expression of *ANK3* significantly enhanced E-cadherin protein expression and inhibited PTC progression, at least in part, by suppression of epithelial-mesenchymal transition (EMT). Our study shows that *ANK3* exerts an anti-oncogenic role in the development of PTC and might be an indolent maintainer of PTC.

## Introduction

Papillary thyroid carcinoma (PTC) is the most common thyroid malignancy, accounting for about 80% of all thyroid cancers, but usually has a good long-term survival (1, 2) . Studies have revealed that ionizing radiation, nodular disease of the thyroid, and family history are risk factors for PTC development, with genetic predisposition to PTC being the most important risk factor compared to other cancers (3, 4). Notably, benign tissues transform into tumor tissues in a complex process that involves many factors, including environmental trigger, loss of tumor suppressor genes, and chronic inflammation (5). Hashimoto’s thyroiditis (HT) is the most frequent autoimmune thyroid disorder and the leading cause of hypothyroidism in the iodine-sufficient areas of the world. The incidence of HT is about 0.3%-1.5% worldwide and the majority of cases occur in women, with the incidence rate in females being about 5-20 times that in males (6).

The association between HT and PTC is always a controversial topic. Clinically, HT often coexists with PTC, suggesting that there may be an immunological association between the two diseases. The association of HT and PTC remains an active focus of research and controversy. Yan et al. reported a higher incidence of HT in BRAF-positive PTC patients, but could not correlate it to prognosis during the relatively short follow-up period (7). However, among patients with diffuse lymphocytic infiltration of PTC, extrathyroidal extension and BRAF-positive mutations were less frequent when serum antithyroperoxidase titers were positive (8). It has been found that serum antithyroperoxidase levels above a certain level are strong indicators of multifocal PTC in patients with HT. Other recent gene expression experiments have demonstrated a strong correlation between immune lymphocytic infiltrates in the thyroid and expression of error-prone DNA repair (9).

However, little is known about the genetic alteration in PTC with HT. In this study, whole-exome sequencing (WES) was performed on 10 pairs of samples obtained from patients suffering from PTC with HT (HT in one lobe and PTC in the other lobe), with the overarching goal of extracting the mutational signatures that cause somatic mutations. Moreover, in the present study, we also revealed that ANK3 expression restrained tumor cell invasiveness and suppressed the epithelial- mesenchymal transition (EMT) of PTC cells through increased E-cadherin expression and it could be an indolent maintainer of PTC.

## Materials and Methods

### Sample Collection

For whole-exome sequencing, we enrolled 10 patients (10 females) suffering from HT in one lobe and PTC in the other lobe, and had undergone surgery at the Eighth Affiliated Hospital of Sun Yat-Sen University from 2017 to 2019. For immunohistochemistry, 40 cases of PTC with HT tissues were obtained from the same hospital in a period ranging from 2009 to 2019. In addition, we explored the expression of *ANK3* using human PTC tissue microarrays consisting of 110 cases of PTC and adjacent normal thyroid tissues also obtained from the same hospital from 2008 to 2020. All of these histological specimens were reevaluated by two experienced pathologists. This study was approved by the ethics committee of the Eighth Affiliated Hospital of Sun Yat-Sen University.

### Immunohistochemistry

Staining for ANK3, NCOR2, BPTF, and PCSK5 were performed using formalin−fixed, paraffin-embedded serial sections. Sections (4µm-thick) were cut from the paraffin blocks and deparaffinized by routine techniques. After microwaving in citrate buffer for 5 min, slides were incubated with anti-ANK3 (1:100, #37413-2, Signalway Antibody, Maryland, USA), anti-PCSK5 (1:150, #47322, Signalway Antibody, Maryland, USA), anti-NCOR2 (1:150, #43109, Signalway Antibody, Maryland, USA) and anti-BPTF (1:100, ab72036, Abcam, Cambridge, MA, USA), and stored overnight at 4°C. Next, sections were incubated with a secondary antibody (Maxim BioCompany, Fuzhou, China). Labeling was monitored by diaminobenzidine (Maxim-BioCompany, Fuzhou, China). Finally, the sections were stained with hematoxylin. ANK3, PCSK5, NCOR2, and BPTF were scored according to the positive percentage and staining intensity of the stained tumor cells. The positive percentage was scored as: 0 = (0−25%), 1 = (26−50%), 2 = (51−75%), and 3 = (>75%). On the other hand, the staining intensity was scored as: 0 (no staining), 1 (weakly stained), 2 (moderately stained), and 3 (strongly stained). If the product of multiplication between staining intensity and the percentage of positive cells was ≥2, it was considered immunoreaction positive (+).

### Library Preparation and Whole-Exome Sequencing

Human genomic DNA was extracted from tissue samples using DNA FFPE Tissue Kit (Qiagen, Hilden, Germany) in accordance with the manufacturer’s protocol. The genomic DNA was then randomly broken to a length of 180-280bp fragments by Covaris. Next, Sure Select Human All Exon V5/V6 kit (Agilent, Santa Clara, USA) was used for library construction and capture experiments according to the manufacturer’s instructions. A total of 334378 exons for 20965 genes were captured by magnetic beads with streptomycin. After DNA quality evaluation, captured DNA libraries underwent sequencing on Illumina HiSeq 4000, which resulted in 150bp paired-end reads from the end of the fragments.

### Exome Sequencing Data Analysis for SNVs and INDELs Calling

It is worth noting that we filtered the raw data obtained from sequencing because it contains adapter reads, low-quality nucleotide, and undetected nucleotides (N), which can significantly influence the analysis of downstream processing. To obtain high quality clean data and adapter reads, reads containing more than 10% of uncertain nucleotides and paired reads when single reads have more than 50% low-quality (<5) nucleotides were discarded. Next, paired-end clean reads were aligned to the reference genome (human_B37) using Burrows–Wheeler Aligner (BWA) (10) and Samblaster (11). If paired reads were aligned to multiple sites, BWA was used to choose the best alignment sites, and if there were two or more alignment sites, any one of them would be chosen randomly. Duplicate results were marked by Samblaster. Moreover, SNP and INDELs were identified and filtered in the Genome Analysis Tool kit (GATK) (12). Based on the SNP (13), variants obtained from previous steps were compared with ANNOVAR (14). Finally, MuTech (15) and Strelka (16) were used to identify SNVs and somatic INDELs in WES data.

### Mutation Spectrum and Mutation Signature Analysis

Cluster analyses were conducted on 96 somatic mutation types using Nonnegative Matrix Factorization (17), whereas mutation spectra were clustered with 30 known signatures on COSMIC (18) to explain the mutation process of samples. Signatures A, B, and C were identified in tumor samples, and their distribution was presented.

### Determination of Significantly Mutated Genes and Screening of Predisposing Genes

Significantly mutated genes (SMGs) were defined as the somatic mutations with higher mutated frequency than the background mutation rate. A SMG test was used to analyze mutations based on tumor samples and a significantly mutated genes landscape heat plot was presented. For SMGs, pathway enrichment analyses were performed using Path Scan software (19). Potential predisposing genes could be identified by detecting germline mutations and comparing to the CGC (20) database.

### Cell Lines, Plasmid Constructs, and Establishment of Stable Cell Lines

B-CPAP and TPC-1 cell lines were obtained from the American Tissue Culture Collection (ATCC), and maintained in RPMI 1640 medium supplemented with 10% fetal calf serum (FCS) and penicillin-streptomycin (Invitrogen, Inc.SanDiego, CA). Human *ANK3* gene (NM_001149) overexpressing lentivirus was provided by Biofavor Biotech (Wuhan, China). Briefly, cells were transfected with the *ANK3*-overexpressing lentivirus (*ANK3*-mCMV-MCS-3Flag-SV40-EGFP) at an MOI of 200 for 24 h. Cells were then selected with G418 (600µg/mL) for three weeks and expanded. Finally, TPC-1 cells that overexpressed *ANK3* were labeled *ANK3*-stably overexpressing TPC-1 cells for animal experiments.

### Flow Cytometry Analysis

The stably transfected cells were harvested, washed with phosphate buffered saline, and subjected to AnnexinV/PI double staining (Invitrogen, CA, USA). The percentage of apoptotic cells was then detected by flow cytometric analysis. The stably transfected cells were collected and washed twice with phosphate-buffered saline, followed by staining with propidium iodide for 30 min (Invitrogen, CA, USA). The cell cycle distribution of the stained cells was assessed on a flow cytometer (BD FACS Calibur).

### *In vitro* Cell Proliferation and Colony Formation Assays

For the cell proliferation assay, 5000 cells were seeded into 96-well plates. Cell viability was assessed for five consecutive days using the Cell Counting Kit-8 (CCK-8) (MedChemExpress, Shanghai, China). For the colony formation assay, 300 cells were seeded into 6-well plates for two weeks, followed by staining with crystal violet and counting the colonies.

### Wound Healing Assay and Transwell Migration Assay

The confluent cell monolayers were wounded by scratching with a sterile 10μl pipette tip and then cultured for 24 h. Cell migration over the scraped area was photographed at 0 and 24 h. At the end of the experiment, the cells were fixed with 4% paraformaldehyde and stained with 1% crystal violet for 15 min.

The cell invasion assay was performed using transwell plates (Costar, New York, USA). Briefly, cells (each at a density of 2×105 cell/ml) were added to the upper chamber with 0.2 mL of serum-free RPMI-1640, whereas 0.5 mL of 10% FBS medium was added to the lower chamber. Cells were then allowed to invade for 48 h at 37°C. After removing the cells on the upper surface of the membrane, cells on the lower aspect were stained with trypan blue.

### Immunofluorescence Staining

Cells grown on cover slips were fixed with 4% paraformaldehyde, permeated with 0.3% TritonX-100, and blocked with 1% BSA. Next, cells were incubated with a mixture of two primary antibodies: rabbit anti-ANK3 (1:100, A20299, ABclonal, Wuhan, China) and mouse Anti-E-cadherin antibody (1:100, ab231303, Abcam, Cambridge, USA), at 4°C overnight. On the next day, cells were incubated with a mixture of secondary antibodies (FITC-labeled goat anti-rabbit IgG or Cy3 labeled goat anti-mouse IgG; dilution, 1:100) in the dark for 1 h. Finally, nuclei were counter stained with 4’,6 -diamidino-2-phenylindole (DAPI, Thermo Fisher Scientific, MA, USA) and images captured using a fluorescence microscope.

### Western Blot Analysis

Cells were lysed in a lysis buffer after washing twice with ice-cold PBS, followed by quantification of the total protein concentrations using a BCA kit (Beyotime Biotechnology, Shanghai, China). Next, 20µg of total protein was boiled for 5 min and resolved using 10% polyacrylamide gel electrophoresis. Proteins were then transferred to a polyvinylidene fuoride (PVDF) membrane. The membranes were blocked in no fat milk and then incubated with primary antibodies overnight at 4 °C. The primary antibodies included anti-ANK3 (1:1000, A20299, ABclonal, Wuhan, China), anti-E-cadherin (1:1000, ab231303, Abcam, Cambridge, MA, USA), anti-vimentin (1:1000, AF7013, Affinity Biosciences, Jiangsu, China), anti-snail (1:500, ab180714, Abcam, Cambridge, MA, USA), anti-twist (1:1000, AF4009, Affinity Biosciences, Jiangsu, China), and anti-GAPDH (1:1000, AB-P-R 001, Xianzhi Biotech, Hanzhou, China) at 37°C overnight. On the next day, membranes were incubated with the secondary antibodies: anti-Mouse IgG-HRP (1:50000, Boster, Wuhan, China) and anti-Rabbit IgG-HRP (1:50000, Boster, Wuhan, China). Finally, the specific protein bands on the membranes were detected using an enhanced chemiluminescence kit (Beyotime, Shanghai, China).

### *In vivo* Mouse Xenograft Study

A nude mouse xenograft model was established using 4-week-old female BALB/C nude mice obtained from Beijing Sibeifu Laboratory Animal Center. Five mice were included in each group. All animal care and experimental procedures were performed in accordance with established guidelines. Briefly, 5×10^6^ TPC-1-ANK3 -GFP and TPC-1-NC-GFP cells suspended in 200µl were subcutaneously injected into the right forelimb armpits of the nude mice, and the tumor diameters for each mouse were measured weekly. After four weeks, mice were euthanized using anesthesia, and the tumors were isolated and measured.

### Statistical Analysis

All statistical analyses were conducted using SPSS 17.0 (SPSS, Inc., Chicago, IL, USA). All data are expressed as mean ± SD. The differences between two groups were compared using Student’s t-test. *P*<0.05 was regarded as statistically significant.

## Results

### Mutational Signatures of PTC With HT

To elucidate the genetics of PTC with HT, WES was performed on 10 pairs of tissues obtained from PTC with HT patients (HT in one lobe and PTC in the other lobe). Cluster analysis showed that samples from the same individual were well matched (**Figure S1**). The average depth of WES ranged from 41× to 579× for HT samples and from 55× to 502× for PTC samples (**Table S1**). This was followed by calling of somatic mutations using the MuTect algorithm. A total of 2272 somatic single nucleotide variations (SNVs) were identified on the exon coding sequence (CDS), including 782 synonymous, 1376 missense, and 89 stop gain, as well as 849 frameshift insertions or deletions (INDELs) (**Table S2**).

**Figure 1:**
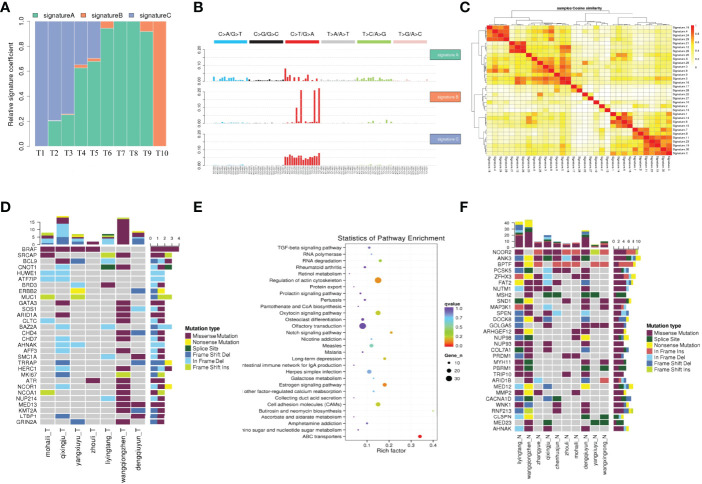
The mutation characteristics of PTC with HT. **(A)** The proportion of each mutation feature in different samples. The abscissa is the sample, and the ordinate is the ratio of each mutation feature. **(B)** Mutation characteristic map. The NMF algorithm was used to cluster 96 mutation types in tumor samples, and the mutation characteristics were obtained. **(C)** Cosine similarity heat map of mutation feature. **(C)** The cosine similarity between the sample mutation features and the 30 known mutation features, the darker the color, the closer the cosine value is to 1, the higher the feature similarity, the more likely it is to be the same feature. **(D)** Landscape map of known driving genes. The abscissa is the sample, the ordinate is the gene, the top is the number of mutations in each gene, and the right is the number of mutations in each gene. **(E)** High frequency mutation gene metabolic pathway enrichment analysis results. **(F)** Thirty SMGs with ≥4 mutations using Samtools in PTC with HT.

To elucidate the mutation characteristics of PTC with HT, we analyzed the mutation spectrum and mutation signature. **Figure 1A** shows that the C>T/G>A substitution was the most common pattern of SNVs in the exon region, which is consistent with the SNVs preference of PTC reported in a previous study (21). Notably, the distinct mutational signatures which were generated from the type and context of somatic mutations may indicate the etiology of cancers (22). The mutations can be divided into 96 types according to the base species at 1bp upstream and downstream of SNVs. Based on the frequency of these 96 types of mutations in tumor, SNVs were divided into different signatures using Nonnegative Matrix Factorization algorithm and then compared to 30 kinds of known mutations on COSMIC. Results revealed close matches to COSMIC signature 5 (found in all cancer types and associated with age or nucleotide excision repair deficiency), signature 11 (exhibits a mutational pattern resembling that of alkylating agents), and signature 30 (whose etiology is still unknown) (**Figures 1B, C** and **Table S3**). All of the three signatures were characterized by predominant composition of C>T/G>A mutation. In addition, signature A was characterized by C>T/G>A displacement at ApCpA trinucleotieds (C is the mutated base), whereas signature B was mainly composed of C>T/G>A mutation at CpCpT, CpCpC, TpCpC, and TpCpT trinucleotieds. Interestingly, the C>T/G>A mutation almost evenly occurred at all 16 types in signature C.

### Significantly Mutated Genes in PTC With HT

Significantly mutated genes (SMGs) in WES samples were identified and 30 known significant driver mutation genes were screened (**Figure 1D**). As expected, *BRAF* was the most significantly mutated gene, which was consistent with previous studies on PTC (23). To further explore how the mutated genes are linked to PTC with HT, we found 30 SMGs with ≥4 mutations (**Figure 1F**) using Samtools (24), most of which have not been well studied in PTC.

Nuclear receptor corepressor 2 (*NCOR2*) is the most frequently mutated gene among all the SMGs that were identified in this study (**Figure 1F**). Located on chromosome 12q24.3, *NCOR2* was previously called silencing mediator for retinoid and thyroid receptor (SMRT) because it was found to bind thyroid hormone receptor and retinoic acid receptor, and inhibit their respective target genes (25). Functionally, *NCOR2* forms a large corepressor complex that contains SI3A/B and histone deacetylases HDAC members, and modulates homeostasis and cancer development (26, 27). Previous studies suggested that decreased *NCOR2* expression promotes breast cancer initiation and progression by acting as a corepressor of estrogen receptor (ER), which has been shown to play an important role in the growth of breast cancer (28). However, to date, no study has shown that *NCOR2* is associated with PTC. In this study, *NCOR2* was mutated in nine of the WES samples, including five missenses, four in frame insertion, and one frame shift insertion.

*ANK3* was also significantly mutated in eight samples, including missense, splice site, in frame deletion, and frame shift deletion. It should be noted that *ANK3* encodes ankyrin G which provides cellular stability by anchoring the cytoskeleton to the plasma membrane. Several recent studies have reported that *ANK3* is strongly associated with bipolar disorder (29, 30). Moreover, studies have demonstrated that *ANK3* is associated with prostate cancer and breast cancer by regulating the stability of AR (31, 32).

Most of the mutation on *BPTF* was in frame deletion. *BPTF*, as a bromodomain PHD finger transcription factor, is critical for epigenetic regulation of DNA accessibility and embryonic development (33). Several studies found that *BPTF* is required for c-MYC transcriptional activity and is associated with EMT as well as tumor progression (34, 35).

The next significant SMG was *PCSK5* (six samples), but its function has not yet been fully elucidated. Several reports revealed that *PCSK5* is involved in the development of embryos (36, 37), whereas only few evidences have confirmed that *PCSK5* is associated with cancer (38).

### Altered Pathways in PTC With HT

The main biochemical metabolic pathways and signal pathways that the SMGs are involved in can be determined by significant pathway enrichment analysis. Herein, Path-Scan was used to analyze the core pathways in which the SMGs in PTC with HT were mainly enriched. The 30 most significantly mutated pathways according to Kyoto Encyclopedia of Genes and Genomes (KEGG) are shown in **Figure 1E**. Results showed that most SMGs were associated with regulation of actin cytoskeleton pathway (***P*** =1.16×10^-2^) (**Table S4**), which has been widely shown to play a critical role in cancer progression and metastasis (39). Notably, some of the well-known oncogenes, such as *BRAF, ITGB1, KRAS*, and *FN1*, all belong to this pathway. In addition, ATP-binding cassette (ABC) transporter pathway showed the most significant correlation (*P*=1.13×10^-5^) and the sequence-alteration of ABC transporter superfamily, including ABCG2, ABCA13, ABCG4, ABCA12, ABCC1, ABCA4, ABCB10, ABCB7, ABCB6, ABCA1, ABCC4, ABCD3, CFTR, ABCC2, and ABCC9 (**Table S4**). ABC genes are essential for cellular functions, and mutations in these genes may contribute to a wide variety of human disorders, including cystic fibrosis, neurological disease, retinal degeneration, cholesterol and bile transport defects, anemia, and drug response (40). Moreover, there is an increasing number of reports indicating that ABC transporters are closely associated with multiple cancers (41).

### Immunohistochemistry Determined the Expressions of *NCOR2, ANK3, BPTF*, and *PCSK5* in PTC With HT

Immunohistochemistry was performed to determine whether a correlation exists between these genetic changes, and the immunoreactivity of *NCOR2, ANK3, BPTF,* and *PCSK5* in 40 paraffin-embedded samples of PTC with HT. Results indicated that *NCOR2* expression was predominantly present in the cytoplasm of PTC cells. However, normal thyroid follicle epithelial cells adjacent to tumor tissues did not stain for *NCOR2*. Among the 40 samples included in this study, 29 scored positively for expression, but only six of them exhibited strong *NCOR2* labeling (**Figure S2**).

Surprisingly, *ANK3* immunoreactivity was detected in all samples (100%, 40/40). Moreover, all samples (100%, 40/40) showed the diffuse strong *ANK3* staining pattern in cytoplasm close to the membrane of PTC cells. Interestingly, ANK3 staining was observed in the colloid of thyroid follicle and cytoplasm of thyroid follicle epithelial cells in HT (**Figure S2**).

BPTF immunoreactivity was detected in 35.0% (14/40) of the samples, of which eight samples had strong *BPTF* labeling. *BPTF* expression was present in the nucleus of PTC cells and normal thyroid follicle epithelial cells adjacent to the tumor (**Figure S2**). In addition, PCSK5 expression was observed in the cytoplasm of PTC cells. However, only four among the 40 samples showed strong PCSK5 immunoreactivity (**Figure S2**).

### *ANK3*, a Novel Diagnostic Marker, Demonstrates Diffuse Strong Immunoreactivity in PTC by Tissue Microarrays

**Figure 2:**
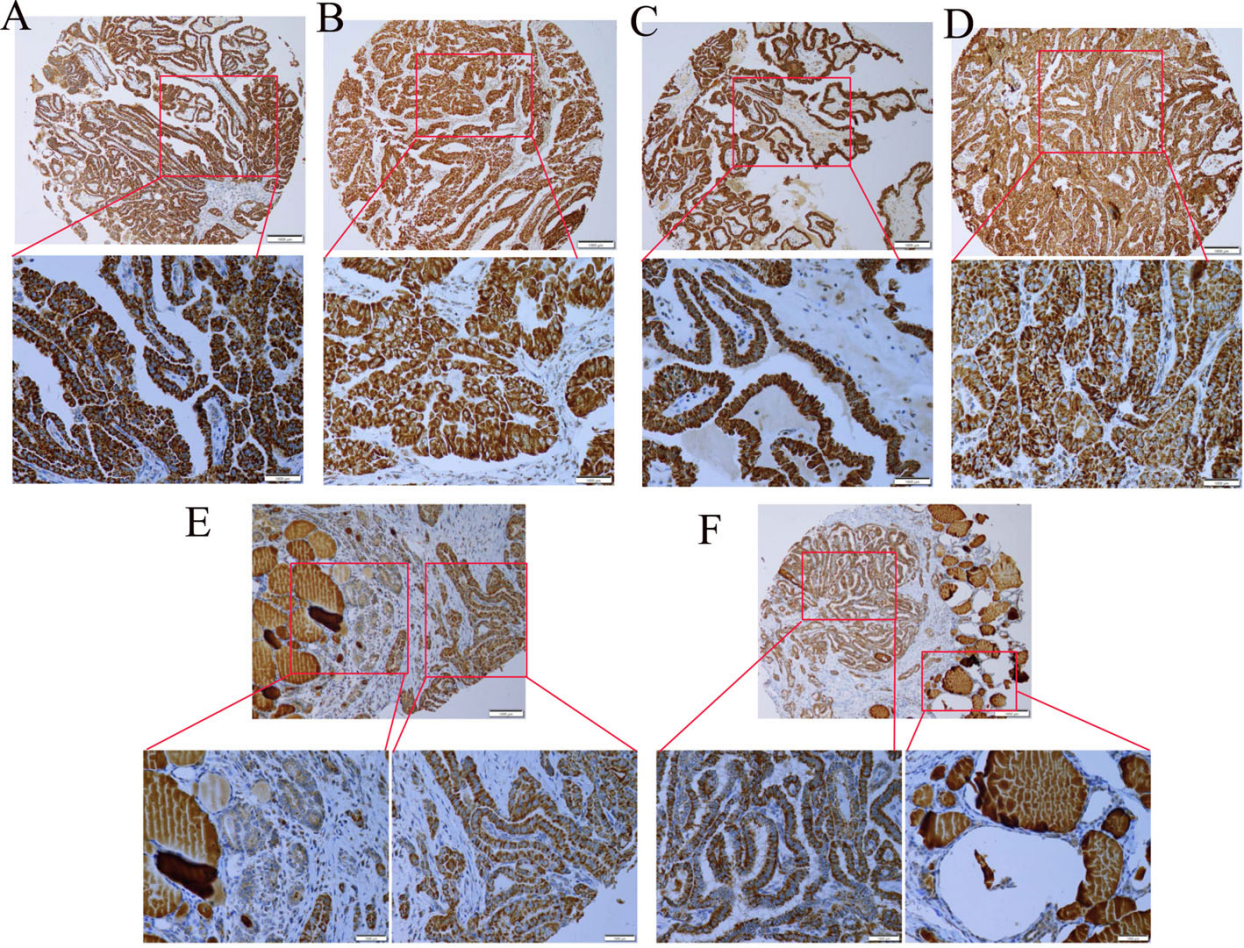
*ANK3* expression in tumour tissues was detected in a tissue array by using immunohistochemistry. **(A–D)** *ANK3* was dominantly expressed in cytoplasm close to the membrane of PTC cells. The above:100×; The below: 200×. **(E, F)** *ANK3* expression in tumour tissues and non-tumour tissues was detected in a tissue array by using immunohistochemistry (100×). Lower figure showed *ANK3* staining has been detected in cytoplasm of normal thyroid follicle epithelial cells and colloid of thyroid follicle. *ANK3* staining was also dominantly expressed in cytoplasm close to the membrane of PTC cells (200×).

To further explore the expression of *ANK3* in PTC tissues, we conducted immunohistochemistry staining for *ANK3* on four PTC microarrays containing 110 pairs of PTC samples and the corresponding tumor-adjacent tissues. It is worth noting that *ANK3* staining had been detected in the cytoplasm of normal thyroid follicle epithelial cells and colloid of thyroid follicle. As shown in **Figure 2**, *ANK3* was predominantly expressed in cytoplasm close to the membrane of PTC cells. Unexpectedly, 108 PTC samples displayed diffuse strong *ANK3*-positive staining in cytoplasm close to the membrane of PTC cells. Only two PTC samples did not exhibit *ANK3* immunreactivity in the peripheral of TMAs, which may be attributed to failure of the reagent to cover the edges of the tissues. Therefore, considering the high positive rate and subcellular localization of *ANK3* in PTC cells, *ANK3* could be a promising new marker for PTC diagnosis.

### ANK3 Suppressed EMT of PTC Cells *Via* Accumulation of E-cadherin at Cell-Cell Contacts

**Figure 3:**
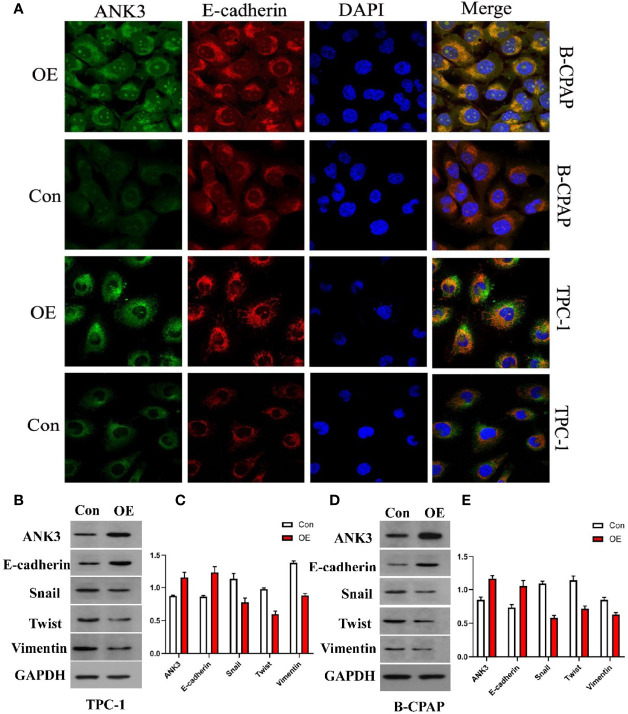
**(A)** Transfection of ANK3 in both B-CPAP and TPC-1 cells significantly increased E-cadherin expression in the membrane of PTC cells by immunofluorescence assay. **(B–E)** Ectopic expression of ANK3 was obviously enhanced E-cadherin protein expression and significantly attenuated vimentin, snail, and twist protein expression in both B-CPAP and TPC-1 cells. (Con: cells transfected with empty vector; OE: cells transfected with ANK3 expression vector).

Kizhatil et al. (42) identified *ANK3* as a molecular partner of E-cadherin, and demonstrated that *ANK3* was required for accumulation of E-cadherin at the lateral cytomembrane in both epithelial cells and early embryos. Therefore, we hypothesized that *ANK3*-mediated suppression of PTC cells migration and invasion was through activation of E-cadherin expression. In the present study, immunofluorescence assay results showed that transfection of *ANK3* in both B-CPAP and TPC-1 cells significantly increased E-cadherin expression in the cytomembrane of PTC cells (**Figure 3A**). Moreover, TPC-1 xenografts overexpressing *ANK3* significantly augmented E-cadherin expression *in vivo* (**Figure 5E**). Next, considering the critical role of E-cadherin in epithelial–mesenchymal transition (EMT) of the malignancy, we used western blot analysis to explore the influence of *ANK3* on the other EMT markers. The obtained results revealed that ectopic expression of *ANK3* significantly enhanced E-cadherin protein expression, and significantly attenuated vimentin, snail, and twist protein expressions in both B-CPAP and TPC-1 cells compared to their respective control cells (**Figures 3B–E**). Altogether, these results suggest that *ANK3* inhibits PTC progression, at least in part, by suppression of EMT.

### Ectopic Expression of ANK3 Exerted an Anti-oncogenic Role Both *In Vitro* and *In Vivo*

**Figure 4:**
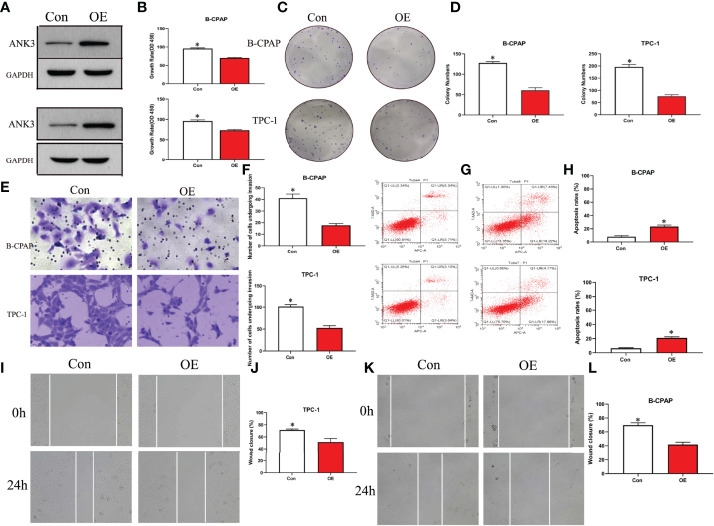
Ectopic expression of *ANK3* exerted anti-oncogenic role in PTC. **(A)** Transfected with *ANK3* expression vectors in B-CPAP and TPC-1 cells, *ANK3* expression was significantly increased compared to the control group (empty vector cells). **(B–D)** Ectopic expression of *ANK3* significantly reduced the cell proliferation and anchorage-dependent growth with MTT assays and colony formation assays. **(E, F)** Ectopic expression of *ANK3* suppressed cell invasion of B-CPAP and TPC-1 cells. **(G, H)** Flow cytometry showed that ectopic expression of *ANK3* increased cell apoptosis. **(I–L)** Ectopic expression of *ANK3* significantly suppressed cell migration of B-CPAP and TPC-1 cells. (*P<0.05, Student’s t-test, n=3 independent experiments).

**Figure 5:**
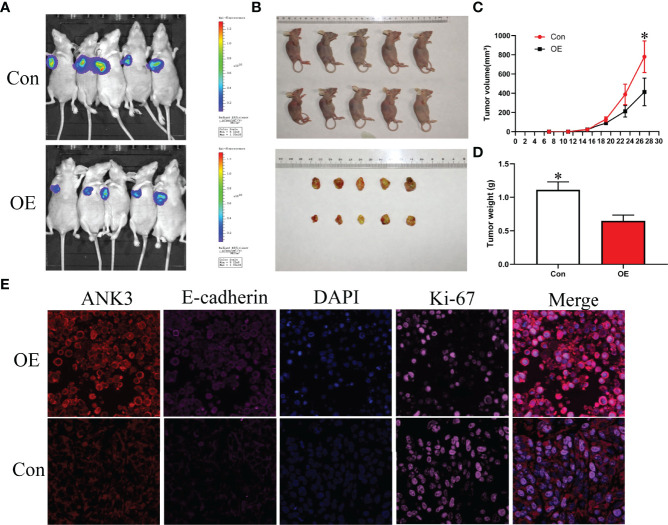
Ectopic expression of *ANK3* suppressed PTC tumorigenesis. **(A, B)** Representative image of tumors in xenograft mouse model. **(C, D)** Over-expression of *ANK3* TPC-1 cells dramatically inhibited tumor growth in the nude mice. Tumor volumes and weight were measured and presented as mean ± SD (n=5 mice per group). P values were determined by the one-way analysis of variance (ANOVA). **(E)** TPC-1 xenografts overexpressing *ANK3* decreased cell proliferation by Ki-67 staining and augmented E-cadherin expression. (*P<0.05).

Gain-of-function study was performed by stably transfecting *ANK3-*overexpression vectors (empty vector CMV-MCS-3FLAG -SV40-Neomycin and CMV-MCS-3FLAG-SV40–Neomycin-*ANK3-* plasmid) into B-CPAP and TPC-1 cells. Western blot analysis results showed that *ANK3* expression was significantly increased in B-CPAP and TPC-1 cells after transfection with *ANK3*-overexpression vectors compared to empty vector cells (**Figure 4A**). Ectopic expression of *ANK3* in B-CPAP and TPC-1 cells markedly decreased their proliferation ability (*P* < 0.05, **Figure 4B**). The colony formation assay showed that overexpressing of *ANK3* in B-CPAP and TPC-1 cells inhibited the colony-forming ability compared to cells receiving empty vector (60.67 ± 6.43 *VS* 127.67 ± 3.21; 76.33 ± 6.11 *VS* 196.00 ± 10.15; *P* < 0.05, **Figures 4C, D**). In addition, flow cytometry showed that ectopic expression of *ANK3* increased cell apoptosis (**Figures 4G, H**). Next, we examined the effects of ectopic expression of *ANK3* on the invasion and motility of B-CPAP and TPC-1 cells. Matrigel transwell invasion and cell migration assays found that ectopic expression of *ANK3* in B-CPAP and TPC-1 cells suppressed their cell invasion (**Figures 4E, F**) and migration (**Figures 4I–L**). To evaluate the effect of *ANK3 Guangzhou in vivo*, TPC-1 cells transfected with control or *ANK3*-overexpression vector were subcutaneously inoculated on the right forelimb armpits of the nude mice. Results showed that mice injected with *ANK3*-stably overexpressing TPC-1 cells exhibited smaller tumor volumes and lighter tumor weights compared to the control group (*P* < 0.05) (**Figures 5A–D**). Moreover, Ki-67 staining showed that TPC-1 xenografts overexpressing *ANK3* exhibited decreased cell proliferation (**Figure 5E**), which is consistent with our cytological observation. Collectively, these results suggest that enhanced *ANK3* expression restrains growth of PTC, thereby exerting an anti-oncogenic role in the development of PTC.

## Discussion

Currently, there is an increased prevalence of PTC in HT patients. Although many studies have suggested that there is a correlation between HT and PTC, the genetic alterations between HT and PTC with HT are still unclear. This study describes the WES of PTC with HT patients. Results showed that the spectra of exome mutation of PTC with HT were consistent with Chinese PTCs, which confirmed the reliability of our WES data (21). Notably, with exception of *BRAF*, a well-known PTC driver gene, this study was the first to identify several genes with high frequency mutations in PTC, such as *NCOR2, ANK3, BPTF*, and *PCSK5*. A previous study reported that *NCOR2*, a nuclear receptor corepressor, serves as a potential drug target in the treatment of breast cancer by interacting with ER, and deficiency of *NCOR2* may cause breast cancer (43). Herein, *NCOR2* was identified as the most frequently mutated gene among the identified SMGs.

Dysfunctions of actin cytoskeleton result in various human diseases. Accumulating evidences have demonstrated that regulation of actin cytoskeleton by multiple molecules may drive cancer motility, EMT and tumor metastasis (39). Most of the significantly mutated genes identified in this study, including *BRAF* and *KRAS*, are widely accepted as prominent oncogenes in PTC (44) and were mainly enriched in the regulation of actin cytoskeleton pathway. Although no study has directly confirmed the correlation between PTC and actin cytoskeleton pathway, several reports have demonstrated that *BRAF* and *KRAS* participate in regulation of actin cytoskeleton (45). Our results suggest that these significant mutations drive HT to PTC probably by regulating actin cytoskeleton signaling.

To determine whether a correlation exists between the genetic changes and the immunoreactivity of *NCOR2, ANK3, BPTF*, and *PCSK5* in PTC with HT, immunohistochemistry was utilized to explore their expression levels. Unexpectedly, results indicated that *ANK3* immunoreactivity was observed in all PTC with HT samples. Next, we further explored the expression level of *ANK3* in PTC by tissue microarrays. Surprisingly, 108 out of the 110 PTC samples displayed diffuse strong *ANK3* staining in cytoplasm close to the membrane of PTC cells. Therefore, given that there is no specific biomarker for PTC, *ANK3* might be a promising diagnostic tool for PTC clinical pathology in the future. A recent study reported that *ANK3* is the top gene associated with mood disorders and stress (46). Researchers had also revealed that the expression of *ANK3* was increased in expression in the amygdala of a mouse model of mood disorders and stress (47).

In the present study, *ANK3* mutation was found in 80% (8/10) of all PTC with HT samples. Moreover, immunohistochemistry confirmed that *ANK3* was expressed in cytoplasm close to the membrane of all PTC with HT cases and in almost all PTC cases. Notably, the expression and localization of *ANK3* was in the cytoplasm of normal thyroid follicle epithelial cells and the colloid of thyroid follicle. Therefore, we speculate that *ANK3* mutation might participate in subcellular localization in PTC cells. In contrast, Kurozumi (31) reported that breast cancer cells showed uniform *ANK3* immunoreactivity primarily in the cytoplasm. These results suggested that the different subcellular localization of *ANK3* protein is associated with different cancer types.

Fortunately, our findings revealed that enhanced *ANK3* expression inhibits growth of PTC cells both *in vitro* and *in vivo*. Recently, Wang et al. (32) reported that *ANK3* knockdown significantly increased cell invasion in prostate cancer cells through an AR-dependent mechanism. This is similar to our finding that overexpression of *ANK3* suppressed migration and invasiveness of PTC cells. One study reported that *ANK3* appeared to be downregulated in several human cancer types, thereby contributing to a poor prognosis (48). Kumar et al. (49) proposed a possible connection between *ANK3* dysregulation and EMT, where the EMT process caused a decrease in the levels of *ANK3* in cancer cells. Durak et al. (50) revealed that *ANK3* loss-of-function increases β-catenin levels in the nucleus, thereby promoting neural progenitor proliferation. Moreover, Kizhatil et al. (42) identified *ANK3* as a molecular partner of E-cadherin and demonstrated that *ANK3* is required for accumulation of E-cadherin. In this study, it was found that ectopic expression of *ANK3* significantly enhanced E-cadherin protein expression and inhibited PTC progression, at least in part, by suppression of EMT. Besides, enhanced *ANK3* expression restrains growth of PTC *in vivo*. Altogether, these findings suggest that *ANK3* could regulate cell proliferation or migration and exerts an anti-oncogenic role in the development of PTC. This study demonstrated that changes in subcellular localization of *ANK3* in PTC cells had important effects on maintaining PTC as an indolent tumor. Increasing E-cadherin expression, ANK3 suppresses the invasion and migration of PTC cells by inhibiting EMT. However, the exact mechanism through which *ANK3* regulates the proliferation of PTC was not elucidated. In summary, this study highlighted the importance of *ANK3* in inhibiting progression of PTC. We also revealed, for the first time, that *ANK3* enhanced E-cadherin expression and attenuated EMT of PTC. Hence, *ANK3* could be an indolent maintainer of PTC and a diagnostic biomarker of PTC.

## Data Availability Statement

The datasets presented in this study can be found in online repositories. The names of the repository/repositories and accession number(s) can be found in the article/**Supplementary Material**.

## Ethics Statement

The studies involving human participants were reviewed and approved by The Ethics Committee of the Eighth Affiliated Hospital of Sun Yat-Sen University. The patients/participants provided their written informed consent to participate in this study. The animal study was reviewed and approved by The Ethics Committee of the Eighth Affiliated Hospital of Sun Yat-Sen University.

## Author Contributions

CZ and MD conceived the project. JL, CD and CZ performed all experiments and constructed the manuscript. LX, HM and YG analyzed and interpreted all the data and support the experimental techniques. LX and SL collected clinical sample and information. All authors participated in preparing the manuscript and approved the submitted and published version.

## Funding

This study was supported by the National Natural Science Foundation of China (No. 81302245).

## Conflict of Interest

The authors declare that the research was conducted in the absence of any commercial or financial relationships that could be construed as a potential conflict of interest.

## Publisher’s Note

All claims expressed in this article are solely those of the authors and do not necessarily represent those of their affiliated organizations, or those of the publisher, the editors and the reviewers. Any product that may be evaluated in this article, or claim that may be made by its manufacturer, is not guaranteed or endorsed by the publisher.

## Supplementary Material

Supplementary Figure 1 Cluster analysis showed that samples of PTC with HT from the same individual were well matched.

Supplementary Figure 2 Expressions of *NCOR2, ANK3, BPTF* and *PCSK5* in PTC with HT. **(A)** Negative expression of *NCOR2* in PTC with HT. **(B)** Positive expression of *NCOR2* in PTC with HT. **(C)** ANK3 staining has been detected in cytoplasm of normal thyroid follicle epithelial cells and colloid of thyroid follicle in PTC with HT. **(D)** *ANK3* was dominantly expressed in the cell membrane of PTC with HT. **(E)** Negative expression of *BPTF* in PTC with HT. **(F)** Positive expression of *BPTF* in PTC with HT. **(G)** Negative expression of *PCSK5* in PTC with HT. **(H)** Positive expression of *PCSK5* in PTC with HT.

Supplementary Table 1 The average depth of WES for 10 PTC samples with HT.

Supplementary Table 2 The mutation characteristics of 10 PTC with HT.

Supplementary Table 3 The mutation mutation signature of 10 PTC with HT.

Supplementary Table 4 Altered pathways in 10 PTC with HT.
