# Supplementary figures and images for: Genetic Alterations in Papillary Thyroid Carcinoma With Hashimoto’s Thyroiditis： ANK3, an Indolent Maintainer of Papillary Thyroid Carcinoma
Source: Front Oncol. 2022 May 12;12:894786. doi: 10.3389/fonc.2022.894786 (PMC9133634; doi:10.3389/fonc.2022.894786)

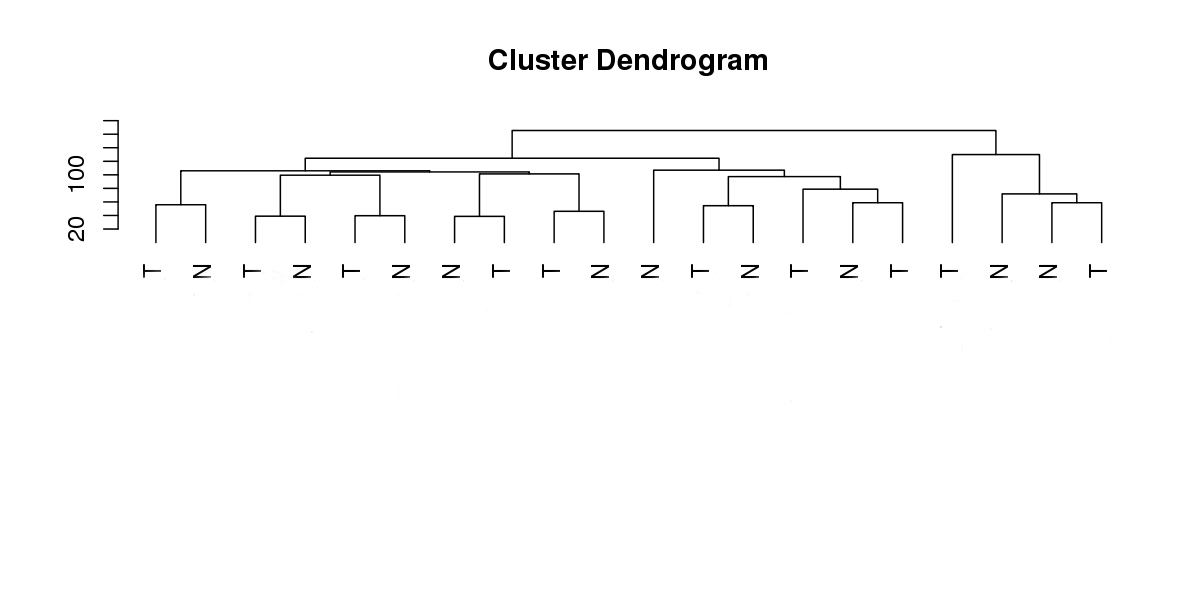

Supplement: Supplementary Figure 1 — Cluster analysis showed that samples of PTC with HT from the same individual were well matched. [file Image_1.jpeg]
